# Dialogue, inclusion, and adaptation in a remote marine sanctuary: evidence from Flower Garden Banks

**DOI:** 10.1007/s00267-026-02515-z

**Published:** 2026-05-29

**Authors:** Kelly H. Dunning, Daniel Morris, Nadia Bowles, Caleb Camus, Frederic Castruccio, Deepak Cherian, Denise Cole, Jaliyl Collins, Tommy Furland, Joanie Kleypas, Kristen Krumhardt, Gretchen Luchauer, Andrea Miranda Paez, Melissa Moulton, Iree Wheeler, Janna R. Willoughby

**Affiliations:** 1 Haub School of Natural Resources and Environment, University of Wyoming 804 E Fremont St, Laramie, WY USA; 2 NSF National Center for Atmospheric Research (NCAR) 1850 Table Mesa Dr, Boulder, CO USA; 3 College of Forestry, Wildlife, and Environment, Auburn University, Auburn, AL USA; 4 Auburn University Minorities in Agriculture, Natural Resources, and Related Sciences, Auburn, AL USA; 5 Applied Physics Laboratory, University of Washington, Seattle, WA USA

**Keywords:** Adaptive management, Climate, Environmental governance, Protected areas, Social-ecological systems, Stakeholder engagement

## Abstract

Interdisciplinary, stakeholder-engaged research is increasingly being used for managing climate change in social-ecological systems. We apply a Collaborative Adaptive Experimental Governance lens to the Flower Garden Banks National Marine Sanctuary, a remote, relatively pristine coral reef system ~130–190 km offshore in the Gulf of Mexico, where biodiversity protection coexists with recreation and offshore energy. We coupled participatory social science with climate and ecosystem modeling to inform dialogue with decision-makers and users. First, we generated scenarios using Community Earth System Model2-LE ocean temperature and aragonite saturation state to characterize warming and acidification; translated heat stress into a variability-based coral bleaching index; and projected demersal and pelagic fish biomass. We then conducted 37 semi-structured interviews (managers, oil and gas, commercial and recreational fisheries, dive operators, Non-governmental organizations, and science/education), employed multi-coder reliability, and triangulated findings with policy and legal documents. Results highlight the centrality of the Sanctuary Advisory Council in structuring inclusive dialogue, co-producing recommendations, and supporting outreach in distant coastal communities. Multi-level coordination among NOAA, the Gulf of Mexico Fishery Management Council, and the Bureau of Ocean Energy Management enabled boundary expansion and reconciled conservation with industry and fishing interests. Key barriers to adaptive responses include offshore remoteness and logistics, limited public awareness, funding constraints, trust deficits, and procedural delays; pressures that intersect with warming, acidification, and episodic hypoxia. Our study shows that remote marine protected areas can operationalize inclusive, experimental governance to align science and management, but sustained investment in monitoring, restoration capacity, boundary-spanning outreach, and cross-agency coordination is needed.

## Introduction

Research that bridges physical, natural, and social sciences is increasingly valued, particularly when it includes the voices of stakeholders, defined as those whose lives are directly affected by environmental change, in the decision-making process. These *participatory* approaches can empower decision-makers and other stakeholders to work closely with scientists to specify what information they need to improve management of a resource, what format that should take, and how the management-relevant questions are answered in practice (Duea et al., 2022; Woolf et al., 2016). To further the usefulness of participatory research to end-users like decision-makers, it is increasingly considered best practice for interdisciplinary teams of scientists from a range of backgrounds to collaborate during a research project, while simultaneously rigorously engaging end-user, and decision-makers (Cheruvelil et al., 2014; Pennington et al., 2013). Collaborative, interdisciplinary research can help support marine protected areas, where the sensitivity of ecosystems and the presence of ecologically and economically important species demand integrated approaches that span ecological, social, and policy dimensions.

One such where collaborative, participatory science can help protect ecosystems undergoing environmental change is the United States marine sanctuary network, which offers federal protection to ecosystems found nowhere else in the world. One of these marine sanctuaries, the Flower Garden Banks National Marine Sanctuary (hereafter “Flower Garden Banks,” “Flower Gardens,” or “the sanctuary”), is one of the world’s most pristine coral reef ecosystems and is the only national marine sanctuary entirely in the Gulf of Mexico (Hickerson et al., 2012; Hickerson and Schmahl, 2005). Flower Garden Banks, established in 1992, protects 17 unique and biodiverse underwater mountains, called “banks,” and compared to other marine protected area coral reef systems, is located quite far offshore, at 130 to 190 kilometers (80-120 miles) away from the states of Texas and Louisiana (Johnson et al., 2019; Office of National Marine Sanctuaries, 2021). The remoteness of this marine sanctuary makes its management unique due to its low visitation numbers, seeing for example approximately 2000 divers per year, making it a much lesser visited marine sanctuary compared to the Florida Keys National Marine Sanctuary which attracts over 700,000 divers per year (Respondent 6). The Gulf of Mexico coastal region is the fastest growing region in the United States, with a growth rate of 26.1% in 17 years (McKinney et al., 2021). The Gulf of Mexico, and waters adjacent to the marine sanctuary, is a major hub for the offshore oil and gas industry, source of much domestic energy production and contributing about $30 billion to gross domestic product (GDP) (Energy & Industrial Advisory Partners, 2023). In the early days of the sanctuary, the oil and gas industry successfully advocated for a smaller sanctuary than was originally proposed to preserve opportunities in one of the most important economic sectors in the state of Texas and its waters. Thus, Flower Gardens presents a unique case of a remote, relatively pristine ecosystem in a region with high levels of growth and industrial economic importance. Our research examines how remote, pristine, unique ecosystems, and the managers and stakeholders that steward them, are adapting to the impacts of environmental change, with a specific focus on opportunities for collaborative, adaptive management arrangements that emphasize including many voices of diverse stakeholders and enabling dialogue.

## Literature review

The banks of Flower Garden Banks are ancient salt deposits, shaped like domes on the seafloor, with varying structures depending on the seafloor depths. The peaks, at depths ranging from 60-130 feet, contain coral reefs that are different from more common Caribbean and Western Atlantic shallow water fringing reefs such as those of the Florida Keys National Marine Sanctuary as they are deeper by an order of magnitude and therefore harder to reach (National Ocean Service, 2025). Boulder corals dominate the warm-water reefs of the sanctuary and provide habitat for numerous species. These species include several that are endangered, such as five species of sea turtle, Rice’s whale, and manta rays. Below these coral reefs is a layer of rubble from sunken reef structures, followed by mesophotic coral reefs, which start at 70 to 80 meters (Rezak et al., 1985). The deeper corals present at Flower Garden Banks are significant ecologically, and to stakeholders, because they provide important habitat used by economically valuable fish species.

Flower Garden Banks is vulnerable to harmful impacts from environmental change, including rising sea temperatures and ocean acidification. Water temperatures have already warmed ~1°C over the past 30 years in this region, and projections from Earth system models indicate that temperatures may warm an additional ~4°C over the coming decades (Alexander et al., 2018). This puts Flower Garden Banks corals at high risk for bleaching, as well as harmful algae blooms, as in the Florida Keys. The Flower Garden Banks, despite being more remote than many marine sanctuaries, experience an additional human-caused stressor: hypoxic (low oxygen) conditions. Hypoxia is driven by excess nutrients in river runoff (most notably the Mississippi River) and is compounded by warming-driven ocean stratification. Hypoxia can lead to massive die-offs of marine life, like the event observed in 2016 during which dying corals, sea stars, urchins, clams, and other invertebrates were found on the seafloor (Le Hénaff et al., 2019).

Flower Garden Banks National Marine Sanctuary provides important recreational and economic opportunities, though access is limited due to its remote location, requiring a seven- to ten-hour boat trip offshore. Stakeholders include commercial and recreational fishers, conservationists, researchers, educators, and oil and gas representatives, while reef users are primarily divers and recreational fishers. Compared to nearshore sanctuaries, visitation is low, public awareness is limited, and governance dynamics are less studied. These factors shape how stakeholders connect with and manage the sanctuary.

Understanding the governance and management of the Flower Garden Banks National Marine Sanctuary requires approaches that integrate ecological dynamics with the perspectives of diverse stakeholders who interact with this remote coral reef ecosystem. Participatory and co-produced research has proven especially valuable in addressing complex social-ecological systems, where local knowledge and interdisciplinary insights can enhance resilience and management outcomes (Eelderink et al., 2020; Walker et al., 2002). Participatory research fosters collaboration between scientists and stakeholders, generating context-specific knowledge that improves both ecological and social outcomes (Snapp et al., 2023). Similarly, knowledge co-production has been identified as a cornerstone of conservation science, ensuring that research is relevant, legitimate, and actionable for decision-maker end users (Norström et al., 2020). However, the benefits of participatory and collaborative approaches are context-dependent and may vary based on the number and diversity of stakeholders, institutional structures, and ecological and social conditions. In the context of climate adaptation and resilience, participatory approaches can help stakeholders at Flower Garden Banks confront challenges such as coral disease, bleaching, and hypoxia, while also addressing underlying governance and equity issues (Parsons et al., 2025). Beyond ecological resilience, participatory research has also been recognized for its role in advancing environmental justice and uncovering structural vulnerabilities that disproportionately affect some communities and stakeholder groups (Davis and Ramírez-Andreotta, 2021). These insights highlight why Flower Garden Banks provides a useful case for examining how participatory, interdisciplinary research approaches operate in a remote and logistically constrained marine system with emerging climate stressors.

Effective governance of marine protected areas hinges on stakeholder inclusion and dialogue. Other studies have shown as much, ranging from participatory planning workshops to perception surveys, coalescing on the idea that stakeholder engagement improves management design, compliance, and legitimacy (Dunning, 2021a; Rasheed and Abdulla, 2020; Rodrigues et al., 2024; Wally et al., 2024). However, benefits are contingent on clear objectives and institutionalized processes, as underscored by Schwermer et al. (2020) and O’Connor et al. (2024). Case studies further illustrate that absent dialogue undermines stakeholder trust limiting conservation and socio-economic links to marine protected areas (Cavalcante de Oliveira Júnior et al., 2021). This evidence highlights how inclusive, transparent, and well-designed participatory frameworks can enhance management within the unique context of the remote Flower Garden Banks.

This study explores governance at Flower Garden Banks with a particular focus on fostering dialogue and inclusion among diverse stakeholders. We examine how different groups engage with the sanctuary, how their perspectives are incorporated into management, and how collaborative processes shape decision-making. Specifically, we ask: (1) In what ways are stakeholders included in governance and decision-making at Flower Garden Banks, and how do dialogue and participation influence outcomes? (2) What challenges do managers and stakeholders face in creating inclusive governance, and how are these addressed through collaborative engagement? Undertaking stakeholder-inclusive research at Flower Garden Banks can help fill a necessary gap in research because its remoteness limits public awareness and direct engagement compared to other national marine sanctuaries. These factors mean that dialogue and inclusion may not occur automatically and may require intentional effort to foster and ensure that management reflects the perspectives of stakeholders. Because inclusive management is often associated with increased legitimacy and stakeholder stewardship, it may support biodiversity conservation and enhance resilience to environmental change in systems such as the Flower Garden Banks.

## Methods

### Study Site

**Fig. 1:**
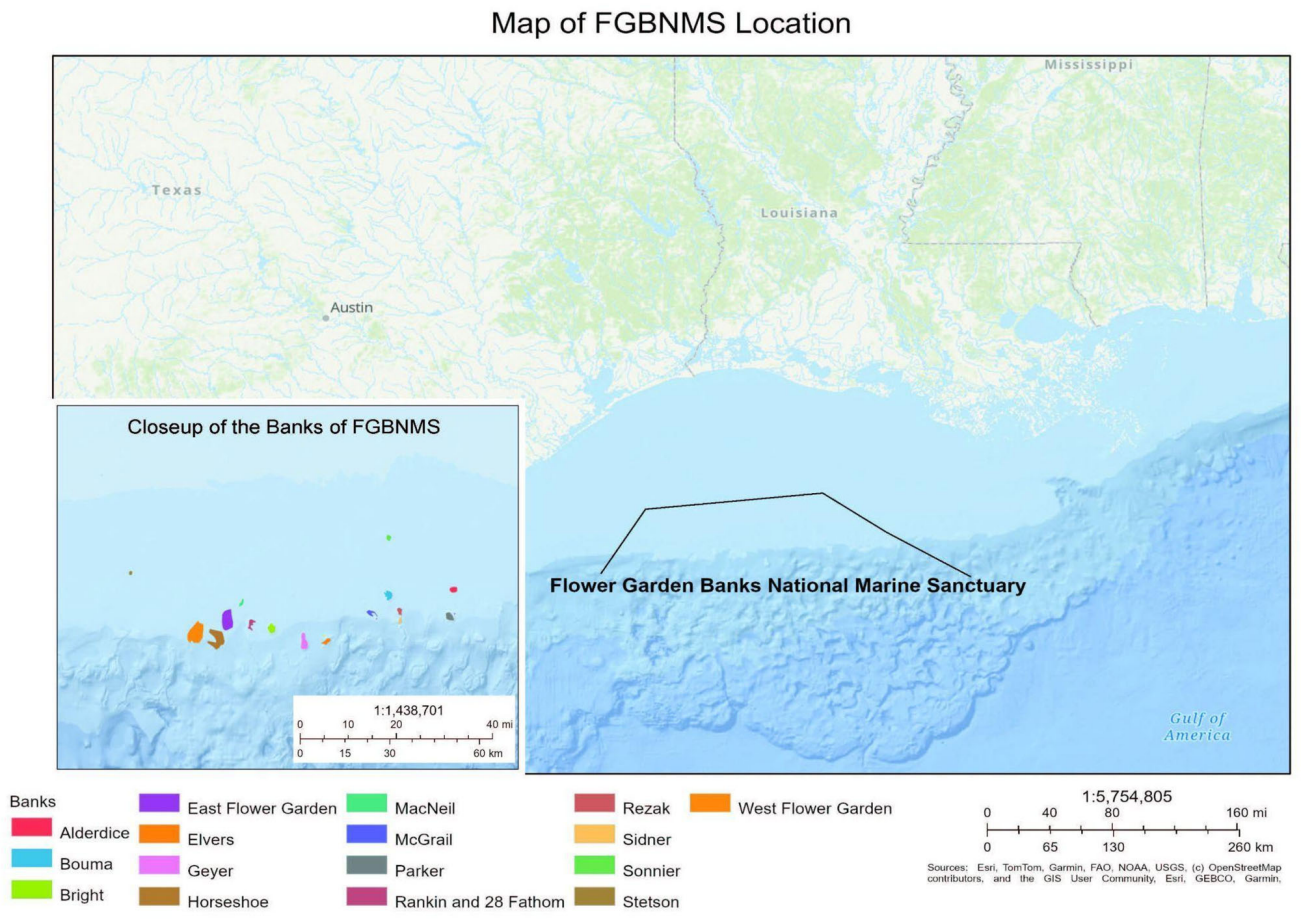
Location of the Flower Garden Banks National Marine Sanctuary (FGBNMS) in the northwestern Gulf of Mexico. The main map shows the sanctuary’s position relative to the Texas and Louisiana coasts. The inset provides a detailed view of the 17 banks protected within the sanctuary. Colored polygons indicate individual bank boundaries

This study took place in the hubs where Flower Garden Banks stakeholders and reef users are based, primarily the Galveston and Houston metropolitan areas due to the location of the local office for National Marine Sanctuaries and in-person Sanctuary Advisory Council meetings. However, Flower Garden Banks stakeholders and reef users can be found from Corpus Christi, TX, to New Orleans, LA, all roughly 100 miles from Flower Garden Banks (Fig. 1).

### Materials and methods

This study builds on established collaborative governance scholarship, particularly the collaborative governance model (Ansell and Gash, 2008), which emphasizes the importance of starting conditions, institutional design, facilitative leadership, and collaborative processes in shaping governance outcomes. Rather than replacing these established approaches, Collaborative Adaptive Experimental Governance is applied here as a complementary extension designed specifically for climate-affected social-ecological systems, where model-based ecological futures and experimental responses become central components of governance (See Table 2 below for additional breakdown of how we built on preexisting theory).

We developed and prototyped participatory research that employs the lens of Collaborative Adaptive Experimental Governance in previous work also on the marine sanctuary system (Morris et al., 2024). Collaborative Adaptive Experimental Governance extends broader adaptive governance and co-production approaches by explicitly linking 1) climate and ecological modeling used to generate plausible future scenarios, 2) structured stakeholder dialogue used to identify governance capacities and constraints in light of those scenarios, and 3) experimentation in management responses, whether implemented or prospective (Morris et al., 2024). What distinguishes the framework is the integration of modeled ecological futures as a deliberate input into participatory governance processes, rather than treating stakeholder engagement and biophysical modeling as separate activities.

**Fig. 2:**
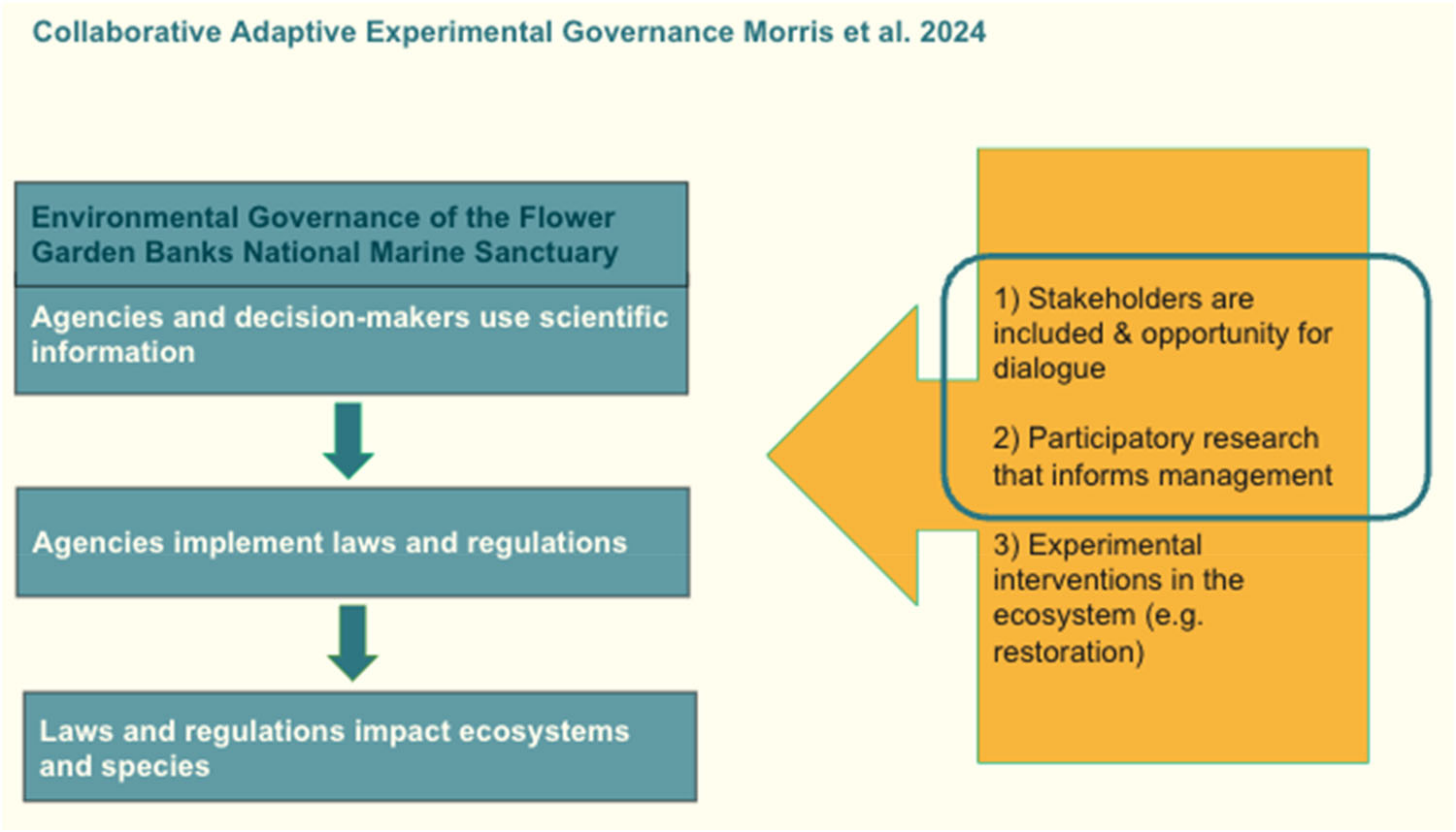
Conceptual framework of Collaborative Adaptive Experimental Governance as applied to the Flower Garden Banks National Marine Sanctuary. Blue-boxed elements depict the theoretical concepts emphasized in this manuscript (1–2), while the orange shaded box denotes future research directions focusing on experimental interventions. Adapted from Morris et al. (2024)

Collaborative Adaptive Experimental Governance has three characteristics: 1) institutions that enable experimental forms of dialogue and inclusion with stakeholders, 2) collaborative and participatory research design that can be used to better understand blockages to policy responses to climate change, and 3) experimental interventions in the ecosystem. In this study, we focus specifically on the first two components, institutions that enable dialogue and inclusion, and the use of scenario-informed interviews, rather than experimental interventions themselves. In this sense, Collaborative Adaptive Experimental Governance can be understood as extending collaborative governance by explicitly incorporating modeled ecological scenarios as inputs to collaborative processes and by emphasizing experimentation under conditions of environmental uncertainty. Figure 2 below illustrates our theoretical framework, with items in blue indicating the theoretical concepts we focus on in this manuscript (concepts 1-2) with forthcoming manuscripts examining experimental interventions in the system. The codebook in Table 1 below defines concepts of our theoretical lens and provides an example of how these concepts were coded for once data were obtained. This manuscript focuses specifically on the first two components of the framework, institutions enabling dialogue and inclusion, and participatory processes used to identify barriers to responding to modeled climate risks. The third component, experimental interventions in the ecosystem, is beyond the scope of this article and is addressed in forthcoming work.

**Table 1:** Operationalization of the two Collaborative Adaptive Experimental Governance components analyzed in this study

Concept that we coded for	Definition and example
Environmental governance	Political actors, agencies, and decision-makers using scientific information to make policies resulting in changes in ecosystems. Any statement from respondents involving political actions for conservation in the sanctuary received this code.
Institutions that enable experimental forms of dialogue and inclusion of stakeholders	Institutions are rules and norms that shape conservation activities in the sanctuary. These can include written management plans, government agencies, and non-governmental organizations that make and follow rules for implementing conservation activities. Statements from respondents that received this code dealt specifically with institutions that enhanced stakeholder inclusion and exchanges with one another to improve understanding.
Collaborative & participatory research process that elicits blockages to policy responses to environmental change	Any statement from respondents that addressed impediments to responding to the climate change scenarios (narrative stories and images) received this code. Any information gained from conversational interviews with decision-makers that invited decision-makers to co-create research questions and priorities also received this code.

We ran a set of climate and ecological models to better understand how environmental change might affect Flower Garden Banks and to communicate these possible changes to stakeholders, asking them what barriers might prevent effective responses. To capture climate drivers that coral reefs are most sensitive to, we used data on ocean temperature and acidification from the Community Earth System Model version 2 Large Ensemble (CESM2-LE) model, a climate modeling framework that produces multiple realizations of Earth system projections under historical and future forcing, allowing researchers to assess both forced climate change and internal variability (Danabasoglu et al., 2020). For temperature, we averaged conditions between 15 and 45 meters to reflect the depth of Flower Garden Banks’ warm-water reefs. For acidification, we calculated the saturation state of aragonite (Ω_aragonite_), a measure of whether corals and other calcifying organisms can build their skeletons; values below one signal corrosive conditions that threaten reef growth (Doney et al., 2009). To translate warming into coral impacts, we applied the variability-based bleaching model described by Teneva et al. (2012), which tracks thermal stress relative to a rolling 50-year baseline and produces a bleaching index indicating whether corals are likely to experience no, moderate, severe, or very severe bleaching in any given year. Finally, to evaluate potential changes in reef fish, we used the Fisheries Size and Functional Type Model (hereafter “FEISTY”) (Krumhardt et al., 2024; Petrik et al., 2019), which simulates how demersal (bottom-dwelling) and pelagic (open ocean) fish communities respond to shifting ocean conditions. FEISTY uses variables such as water temperature, food availability, and zooplankton dynamics to project biomass across different fish size classes. We analyzed changes in demersal and pelagic biomass from 1990 through 2060. These model-derived scenarios were used as structured prompts during interviews to elicit stakeholder perspectives on potential responses to projected environmental change.

**Fig. 3:**
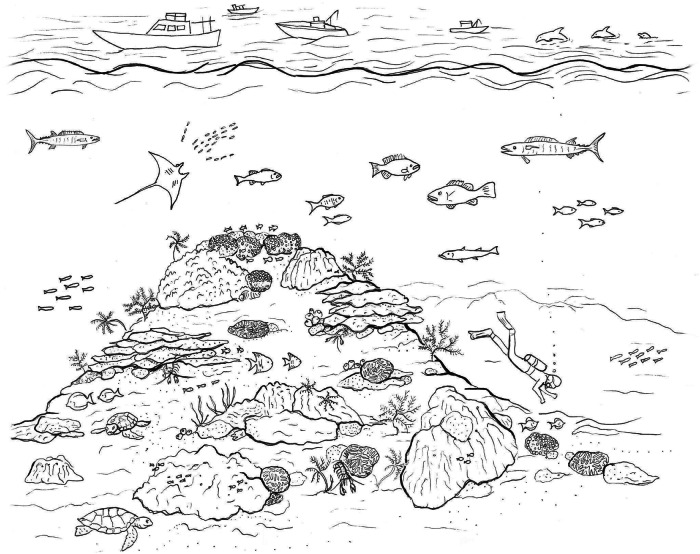
By the year 2043 (20 years from now), there will be a moderate coral bleaching event at Flower Garden Banks at least once every year, which means that the reefs will be under stress and a percentage of the coral will die, especially at the peaks of the banks, affecting the fish that use the reefs as habitat. Most remaining corals will be broken or diseased and will be noticeably less colorful. The surviving corals will not be able to replace broken or damaged coral fast enough, and the structure of the reef will appear greatly diminished. Fish biomass will decrease, and important species that use the reef as habitat/home like greater amberjack, snapper, and grouper will be noticeably less present. Even with this scenario, however, it is possible that some corals will be more heat tolerant, and those will be the ones you will start seeing more frequently. What are your options to respond? (If respondents needed more information, we’d estimate coral mortality at 50%)

**Fig. 4:**
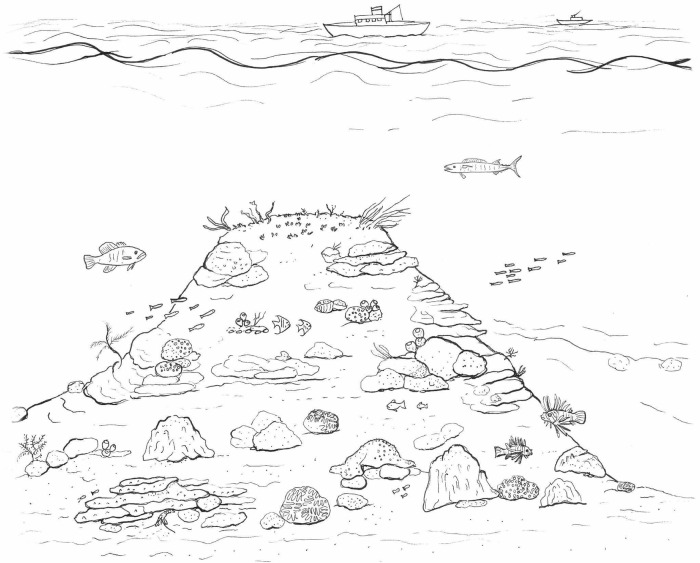
Flower Garden Banks as projected 20 years into the future. Illustration provided by Kristen Krumhardt

It’s important to note that the modeled climate scenarios were not included as background context alone, but were used as structured elicitation devices within interviews (Appendix 1; Figs. 3–4). Respondents were presented with a scenario describing projected warming, recurring bleaching, and declines in fish biomass by approximately 2043, and were then asked to respond specifically to what management options were possible and what barriers might constrain responses. In this way, the scenarios served to anchor stakeholder dialogue around a common set of plausible future conditions, rather than eliciting only general perceptions of environmental change.

To further situate findings in relation to established collaborative governance theory, we also mapped emergent findings against core dimensions of collaborative governance (Ansell and Gash, 2008) and Collaborative Adaptive Experimental Governance (Table 2).

**Table 2:** Alignment of study findings with collaborative governance and Collaborative Adaptive Experimental Governance dimensions

Finding	Collaborative Governance (Ansell and Gash, 2008)	Collaborative Adaptive Experimental Governance	Evidence from Flower Garden Banks
Long-standing stakeholder relationships	Starting conditions	Institutions enabling dialogue and inclusion	SAC relationships, oil & gas coordination, fisheries collaboration
Sanctuary Advisory Council	Institutional design	Institutions enabling dialogue and inclusion	SAC representation, working groups, recommendations
Joint fact-finding and boundary expansion	Collaborative process	Participatory processes	Boundary Expansion Working Group, BOEM coordination
Climate scenario elicitation	(Less explicit in original model)	Participatory processes informed by modeled futures	Scenario-based interviews on bleaching/fish decline
Constraints to adaptive response	Governance constraints (implicit)	Blockages to policy response	Funding, remoteness, bureaucracy, trust
Prospective restoration/monitoring responses	Adaptive outcomes (partial)	Experimental interventions (prospective only)	Restoration readiness, monitoring demand

The interview guide contained ten core questions, typically requiring about 15 minutes to complete, though many conversations extended over an hour. Because communities connected to the sanctuary are geographically dispersed, we conducted a total of 37 interviews between July 2023 and June 2025: 14 via Zoom; 4 asynchronously remote through an online questionnaire on the Qualtrics platform; and 19 in person (all using the same question manual available in Appendix 1). A semi-structured interview format was chosen to balance consistency across themes with the flexibility to allow participants to emphasize their own priorities and experiences. This flexibility was particularly important in a collaborative project, as it supported shared meaning-making and positioned participants as co-creators of knowledge rather than simply research subjects (Kvale and Brinkmann, 2009).

We used purposive, theoretically informed sampling (Suri, 2011) to identify stakeholders who either directly influence decisions about Flower Garden Banks or play an advisory role. As shown in Table 3, participants included federal and state managers, sanctuary staff, non-governmental organizations, commercial and recreational fishing businesses, recreational diving operators, and educational or scientific institutions (e.g., aquariums, universities). When individuals spanned multiple categories, they were asked to identify the role most central to their professional identity. All participants were recruited under our Institutional Review Board (IRB) exempt protocols, provided with consent forms, and interviewed or given the online survey link.

**Table 3:** Breakdown of respondents, Stakeholder Interview Sample and Triangulation Sources

Stakeholder Category	Number of Respondents (*n* = 37)	Key Documents Reviewed for Triangulation
**Sanctuary decision-makers** (e.g. federal or state agency staff, sanctuary personnel)	4	Sanctuary Advisory Council meeting notes; federal legal mandates including the National Marine Sanctuaries Act (16 U.S.C. §§ 1431 et seq.), Magnuson-Stevens Fishery Conservation and Management Act (16 U.S.C. §§ 1801 et seq.), Endangered Species Act (16 U.S.C. §§ 1531 et seq.), and Marine Mammal Protection Act (16 U.S.C. §§ 1361 et seq.); Texas state regulatory codes (Texas Parks and Wildlife Code, Title 5; 31 TAC Chapter 57)
**Oil and gas representatives**	4	Industry statements, letters, and online communications outlining relationships with the sanctuary
**Commercial fishing operators**	5	Federal fisheries regulations (Magnuson–Stevens Act, 16 U.S.C. §§ 1801 et seq.); Texas licensing and fishing rules (Texas Parks and Wildlife Code, Title 5; 31 TAC Chapter 58); Gulf of Mexico Fishery Management Council materials
**Recreational fishing businesses** (e.g., charter captains bringing clients to the sanctuary)	2	Federal conservation legislation including the National Marine Sanctuaries Act, Magnuson–Stevens Act, Endangered Species Act, and Marine Mammal Protection Act
**Recreational diving operators**	12	Sanctuary permits restricting anchoring/mooring and prohibiting damage to resources; U.S. Coast Guard vessel inspection standards (46 CFR Parts 1–199); Texas vessel safety and environmental rules (Texas Parks and Wildlife Code, Title 4; 31 TAC Chapter 55)
**Educational and scientific institutions** (e.g., aquariums, universities)	10	Strategic and programmatic planning documents relevant to sanctuary initiatives

To enhance the rigor of our qualitative analysis, we employed multi-coder reliability, with multiple researchers independently coding interview transcripts and then reconciling differences through discussion to ensure consistency and validity in theme development (Miles and Huberman, 1994). In addition to two coders analyzing interview transcripts, we also used policy documents listed in Table 3 to triangulate interview data to ensure that perceptions mirrored realities of governance. These 12 policy documents included federal regulations, enabling legislation, conservation plans, and statements from policymakers, non-governmental organization websites, and private actors engaged in sanctuary management. These documents were gathered from sanctuary websites, partner organizations, and in some cases directly from field site visits.

Interviews were transcribed both in real time and shortly after the interview, with confidentiality or respondents ensured to safeguard confidentiality in the small, closely connected sanctuary community. To validate and contextualize the interview data, we also reviewed sanctuary policy documents, such as advisory council meeting minutes, a second time prior to write up of the results. Interviews concluded when we reached saturation, when responses became repetitive and no new themes emerged, at 37 interviews (Guest et al., 2020; Mason, 2010). Together, these multiple data sources provided a more robust picture of stakeholder perspectives and governance dynamics.

In qualitative research, expectations can be used instead of hypotheses. In a single case study, the variables of theoretical interest are where we will expect thematic patterns of interest to emerge from respondents. Specifically, we expect to see patterns within the sanctuary emerge regarding stakeholder inclusion, dialogue that impact management techniques and ecological outcomes. We note that our sample included a relatively higher representation of respondents from academic and recreational diving sectors. As a result, the perspectives captured may be shaped by these groups and may not fully reflect the views of all stakeholder categories within the sanctuary system.

## Results

Our results are organized around key themes emerging from stakeholder interviews, including governance structure, institutions supporting dialogue and inclusion, collaborative policy outcomes, and barriers to climate adaptation.

### Governance Structure and Key Actors

The most important institutions governing Flower Garden Banks begin with the National Marine Sanctuaries Act, originally passed in 1972, which controls the way that sanctuaries are created and managed due to their unique ecology or cultural importance. Through this law, the National Oceanic and Atmospheric Administration (NOAA) has the authority to make regulations that govern the sanctuary. One of the most important parts of this law is the requirement that management plans are reviewed every 5 years and that public input is considered when revising the management plans. Public engagement and education programs are also enabled via this law (Respondent 6). There are also several laws that ensure species-specific conservation in the sanctuary, including the Endangered Species Act for threatened and endangered species; the Marine Mammal Protection Act, for whales and other marine mammals; and the Magnuson Stevens Fishery Conservation and Management Act, which protects recreational and commercial fisheries in federal waters.

Our interviews showed that the main stakeholders groups include (1) those concerned with protecting biodiversity (e.g. NOAA, the federal sanctuary system including its local office and the national office in Washington, D.C., conservation non-governmental organizations, and educational institutions like universities and aquariums), (2) the oil and gas industry, (3) commercial fisheries, and (4) tourism and recreation stakeholders (recreational anglers and divers). Also included are the local communities that line the coasts closest to the sanctuary, which include Galveston, Freeport, Port O’Connor, and Port Aransas (Fig. 1). Additional agencies at the federal and state levels are involved with management, as interagency coordination is required in sanctuary management plans. In Flower Gardens, these include the state fish and wildlife agency, Texas Parks and Wildlife, which is concerned with fisheries in state waters, as well as the Bureau of Ocean and Energy Management, which regulates offshore oil and gas.

### Institutions Supporting Dialogue and Inclusion

The most important institution that enables dialogue and ensures inclusion among stakeholders is the Sanctuary Advisory Council (SAC). Seats on the SAC are awarded based on stakeholder representation, with inclusion of all relevant stakeholders carefully considered when creating these 16 seats for 8 stakeholder groups (Respondents 6-7). The SAC is a formal federal advisory body whose recommendations are advisory to NOAA managers, consistent with the Federal Advisory Committee Act (FACA), which governs transparency, stakeholder representation, and public accountability in federal advisory processes. Its significance lies not in being advisory per se, but in how this structure provides an institutionalized forum through which diverse stakeholders deliberate, generate recommendations, and inform sanctuary decision-making.

Many respondents noted that the SAC makes significant efforts to ensure inclusion of all stakeholder groups through long-standing relationships characteristic of this relatively small sanctuary. The SAC ensures stakeholder views are heard by those with decision-making power, through collaborative meetings, working groups, the publication of letters on behalf of different stakeholders, and joint fact-finding efforts assessing scientific, technological, or management needs of the sanctuary for a variety of decisions (Respondent 22). Efforts to ensure maximal stakeholder participation include hosting video options for busy participants spread out across hundreds of miles of coastline and having designated sanctuary decision-makers to serve as a liaison with the SAC and the sanctuary. These efforts ensure that the SAC’s recommendations are genuinely received, reviewed, and used to make decisions. “The SAC doesn’t make decisions that are filed in a spreadsheet and ignored. We genuinely feel, across groups, that they hear us, and they make hard decisions to ensure most people are happy” (Respondent 11).

The SAC works closely with the sanctuary to advise management decisions but also to assist in public outreach and education events, thus sharing some of the responsibilities required by law of sanctuary staff. Nearly every respondent noted that the sanctuary’s offshore distance and limited public awareness make onshore outreach and education essential. The SAC supports sanctuary decision-makers through engagement activities such as maintaining stakeholder email lists broken down by stakeholder category, an important channel of communication for such an expansive group of stakeholders along the coast. “The SAC increases the capacity of what the managers of the sanctuary itself are capable of doing, and it has a high degree of ownership among stakeholders themselves” (Respondent 19). Two important forms of public awareness building performed by the SAC included public lectures and website maintenance. The SAC helps to host popular public lectures known as “Seaside Chats,” which are a key method to keep coastal residents aware of conservation activities in the sanctuary, and were discussed as one of the main ways coastal residents stayed connected to the sanctuary. The SAC also ensures a public-facing website where nearby communities can learn more about the sanctuary (Respondent 7, Respondent 21).

Many respondents noted the need for more education in the communities along the Texas shoreline, which generally do not see themselves as communities adjacent to a major marine sanctuary because it is so far offshore. In the words of one respondent, “People care more about an issue when it impacts them. People in Galveston don’t necessarily see themselves as living next to a major federal sanctuary, even though they do. This disconnect highlights how the sanctuary’s remoteness complicates efforts to foster stakeholder awareness and inclusion, even in densely populated and industrialized coastal regions. We need to change this perception for people to care enough to support future experimental responses to climate change that might cost money” (Respondent 2, Respondent 1). Similarly, another respondent noted that “It’s surprising how many people don’t know the Flower Gardens exist, so getting more people involved […] on a community level and getting [people] aware that these ecosystems are even out there and could be in danger in the future.” (Respondent 5). Another noted that there is a “lack of connection from the general public. You go offshore here at the beach in Galveston and put your toes in Gulf of Mexico water. But that is not national marine sanctuary water [which is further out]. So there’s that lack of connection for the general public. If you can’t see and touch it, it is out of sight, out of mind.” (Respondent 6). A unique way managers are making the public more aware of the Flower Gardens are novel traveling exhibits, such as the cruise terminal in Galveston, Texas, which is one of the most popular cruise disembarkation terminals in the United States. This exhibit allows cruise customers to see the unique reefs of the sanctuary, and to feel more connected to this reef (Respondent 8). Many noted the need for additional education programs in K-12 schools perhaps in multiple languages given the large Hispanic population in Texas so that communities of all backgrounds can be included in sanctuary stewardship.

Managers spoke of the challenges in even conceptualizing new types of relationships with coastal communities that range in size from small seaside resorts, to major cities, to rural villages. Engagement is challenging in a homogenous community, but Flower Gardens are adjacent to diverse communities that look quite different from one another. Thus, varying sanctuary managers have established engagement activities with groups ranging from K12 schools to farmers markets to make coastal residents feel more like they live “within” a marine sanctuary, and to bring the marine sanctuary more into the local culture (Respondents 1-2). Respondents noted the need to make inroads popularizing the idea of the “blue economy” or the sanctuary economic activity generated by fishing and diving tours, museums, and other experiences tied to the sanctuary (Respondent 4). “ [This] is why our sanctuary aquarium partnerships are so important. When people go to an aquarium and see an exhibit of the flower gardens, that is a sense of connection because most people will never see the Flower Garden Banks. That’s just the reality. You’re not getting out there unless you’re a diver or a fisher. The connection aspect is challenging for us” (Respondent 6).

### Collaborative Policy Outcomes: Sanctuary Expansion

An example of collaborative sanctuary policy-making, where policies are made with high levels of input and inclusion of stakeholders, was the expansion of Flower Gardens to include more habitat and biodiversity (Federal Register, 2021). A public engagement process initiated in 2007 by the SAC, focused on adding new “banks” or unique coral reef ecosystems, resulting in new sanctuary boundaries shaped by stakeholder participation (Respondent 7). The 2021 expansion redrew the boundaries to include 14 additional reefs and banks, or 104 square miles of additional protected habitat (Respondent 7). Over 15 years of developing the specifics of the expansion, experts, stakeholders and the general public were asked to provide input and several public comment periods were solicited prior to the expansion following the federal publication of the environmental impact assessment on the Federal Register (NOAA, 2021). Stakeholders across two groups could have potentially stopped expansion: oil and gas, and commercial fisheries. Instead, a collaborative process set in place by sanctuary managers and the SAC was adopted, which ensured expansion could occur with all groups’ buy-in.

The powerful oil and gas industry, one of the most important economic uses in the Gulf, could have prevented expansion, but instead opted into a collaborative process. Respondents suggested this collaboration did not emerge solely from the expansion process itself, but was built on long-standing formal and informal relationships among sanctuary managers, regulators, and industry actors. These included repeated interactions through advisory processes, alignment with pre-existing legal and regulatory structures, and norms of trust and reciprocity developed over time. These relational foundations appeared to shape the willingness of stakeholders to engage collaboratively rather than adversarially. The collaborative sanctuary expansion process collected input from stakeholders in the oil and gas industry to ensure that activities would not conflict with resource extraction. Between 2016 and 2018, the SAC established a Boundary Expansion Working Group that met over 20 times, working on expansion and pre-existing regulations which could conflict with expansion. The Boundary Expansion Working Group consulted with the Bureau of Ocean Energy Management (BOEM) to ensure BOEM’s “No Activity Zones” overlapped as much as possible with the planned sanctuary expansion. No Activity Zones are areas where oil and gas operations (e.g. drilling wells, anchoring, or laying pipelines) are already restricted to protect ecologically sensitive habitats. With sanctuary managers, the SAC, oil and gas industry stakeholders, and BOEM decision-makers all working together, expansion could be done in a way to not alienate industry stakeholders, ensuring that sanctuary expansion plus the preexisting No Activity Zones did not restrict oil and gas operations to such a degree as to invite lawsuits (Respondent 6). A representative quote from one group of oil and gas practitioners is as follows: “We have always been longtime supporters of the sanctuary. They engage us early and often. They ask us for input when really restrictive regulations might be used, decisions are made together” (Respondent 16). Oil and gas respondents and policy documents noted additional points of collaboration between the sanctuary and the industry beyond collaborative governance that helped solidify the relationship. The sanctuary partners with the industry on monitoring and research, which solves some of the problems with the remoteness of the sanctuary. “The sanctuary can take an entire working day to get to, and if we miss the weather window, we will be canceled. Partnering with oil and gas has helped with monitoring water quality, logistical support like vessel access, and coordination, to expand sanctuary capacity” (Respondent 7). These collaborative arrangements are likely supported by long-standing institutional relationships, regulatory frameworks, and repeated interactions among stakeholders, which may facilitate trust and coordination in multi-stakeholder decision-making processes. The effectiveness of this arrangement may therefore reflect not simply stakeholder inclusion at the moment of expansion, but path-dependent institutional relationships in which prior coordination among NOAA, BOEM, and industry reduced conflict and made negotiated coexistence possible.

Commercial fishermen could have also halted expansion, but instead opted into a collaborative process facilitated by the SAC. Under the Magnuson-Stevens Fishery Conservation and Management Act, one of the 8 regional fisheries management institutions had to provide approval for sanctuary expansion, in this case, the Gulf of Mexico Fishery Management Council (“Gulf Council”), which develops fishery management plans for the Gulf, sets catch limits, and ensures that essential fish habitat is protected. The Gulf Council also ensures stakeholder participation in any policy that it implements. Stakeholders from fisheries had several concerns regarding the expansion, which included possible jurisdictional confusion over overlapping rules between the sanctuary and fisheries policies, potential restrictions on fishing in expansion areas that might interfere with earnings, and disagreements over limitations on certain types of fishing or gear, worrying they could be banned (e.g., trawls, bottom longlines, and dredges). Commercial anglers do not land substantial amounts of catch within the sanctuary because there are gear restrictions of hook and line only. The outcome of the collaborative process was that expanded boundaries were set in ways that enabled sustainable fishing in areas in and around the sanctuary (Respondent 13). Our respondents from commercial fisheries displayed immense knowledge of the importance of protecting the habitat and the quality and abundance of their target species. “Our operation depends on a healthy habitat; the sanctuary provides that. They gave us ample opportunity for input and didn’t just ban fishing outright. (Respondent 18). Similar dynamics were evident in fisheries, where collaborative participation appeared rooted not only in the expansion process itself, but in longer-standing interactions among fishers, the Gulf Council, and sanctuary managers under existing fisheries governance arrangements.

### Barriers to Climate Adaptation

Responses regarding barriers to adaptive responses emerged specifically in reaction to the modeled future scenarios presented during interviews. Across stakeholder groups, the scenarios elicited discussion of constraints associated with offshore remoteness, funding limitations, institutional complexity, and public awareness, suggesting that modeled futures helped focus discussion on concrete governance challenges tied to projected climate risks. This section synthesizes key themes that emerged from stakeholder interviews regarding barriers to responding to projected environmental change. Across interviews, the sanctuary’s offshore remoteness consistently emerged as a defining constraint shaping stakeholder engagement, access, and the feasibility of management responses. Respondents identified five primary blockages to adapting to environmental change: (1) physical distance out to the sanctuary, (2) lack of public awareness about the existence of and the conservation issues within the sanctuary (described earlier), (3) shortfalls of funding, (4) lack of public trust in government, and (5) bureaucratic slow downs inherent to the collaborative governance process.

Respondents noted that the most important factor limiting their possible responses to environmental change is the distance of the sanctuary, located approximately 100 miles offshore. “So when you have a site 100 miles offshore and deep, there’s not a lot you can do in reality. You would need an army of divers to be out there, and we would need a lot of money to be there” (Respondent 6). Many respondents noted that implementing future reef restoration efforts would not only be expensive to reach the reef when considering the frequency needed to respond to anticipated bleaching events, but also to collect data for managers. “It is $10,000 a day for sanctuary staff to be offshore. So, to do a five-day cruise, the sanctuary staff needs $50,000. There isn’t a lack of interest. Everybody’s always interested in the Flower Gardens and wants to know how the corals are doing. It’s the accessibility of the site. It’s expensive to be out there and inconvenient to go 100 miles offshore.” (Respondent 6). Likewise, stakeholders’ research or tourism seasons may shift altogether with environmental change, which might change the ability to access the sanctuary altogether. “If there are stronger storms and different climate patterns, we might not be able to get out there as much. Or, you know, our season could shift. Right now, our best month to be out there is August, but that could change.” (Respondent 5, 6, 7). Many aspects of current research cruises, the most important tool available for managers to collect data and implement responses to climate change, are oriented to accommodate the hurricane season. In the words of one respondent, “It takes 7 hours for our vessel […] to get to Flower Garden Banks. It is a flagship vessel of the National Marine Sanctuary fleet, but it is 15 years old and breaking down, so we have mechanical difficulties” (Respondent 7). With possible shifts in sea surface temperatures and possible increases in hurricanes in the Gulf, long, expensive cruises will become more constrained.

Stakeholders also noted that challenges with funding span the entire federal marine sanctuary system: “It’s a sanctuary-wide thing; it’s not just [Flower Gardens]. If you compare national parks to sanctuaries, parks get so much more money, and [Flower Gardens] gets barely a fraction of what they get, which makes some sense. Obviously, parks are a bit more accessible […] without that funding, [managers] can’t go out and do science, can’t respond to anything, can’t do our research” (Respondent 5). Respondents and budget documents confirmed that the federal marine sanctuary budget in the U.S. is approximately 1% of that of the National Park Service, the federal agency tasked with managing America’s most ecologically and culturally important protected areas on land (Interview 19, NOAA, 2025). Funding challenges were linked to environmental change, with the largest thermal stress events in Flower Gardens coinciding with the time of year when securing extra funds to respond to bleaching events is most difficult: the end of the fiscal year for the federal government (Respondent 7). Thus, even if there’s a major disease outbreak or a bleaching episode where corals need to be relocated, asking for additional funds is almost impossible due to the timing of the end of the fiscal year.

Stakeholders also noted a general lack of trust in the government acting as a blockage to responding to change. One way that managers have overcome this lack of trust is by outsourcing some community outreach and education to nearby aquariums. “The general public trusts messaging at aquariums more than the government. So, people have a lot of faith in zoos and aquariums. So partnering with them is an excellent way to get the message out.” (Respondent 6).

A final potential blockage to responding to environmental change is the layers of bureaucracy, which may have overlapping or competing rules regarding subjects such as fisheries management or closures of different fisheries within and between Flower Garden Banks and the larger Gulf of Mexico systems. Likewise, getting new rules in place is time-consuming, particularly for collaborative processes, meaning reacting to rapid change may not be possible. The collaborative process to enact a wahoo management plan, for a popular recreational sport fish, is intended to grapple with some of these regulatory or jurisdictional overlaps and make rules clear to anglers in the sanctuary. “We formed the wahoo working group so that it would not take 15 years to come to an agreement about our recommendations” (Respondent 12). One stakeholder notes the challenge of responding to change in a timely manner: “Management gets hard and tricky because you have to go through so many levels to change things […] getting [new rules] in place in time to respond to rapid change is tricky.” (Respondent 5). One respondent noted the process for collaborative decision-making can involve working through a decision in a working group, then the SAC then approaching the sanctuary superintendent, then managers in NOAA, then managers at the highest levels of sanctuary headquarters. “Where does it go from there? The governor? It’s a very long process, which is unfortunate because a lot of times when it comes to environmental change, it can be, you know, rapid in happening” (Respondent 17, Respondent 5).

## Discussion

Our research examined collaborative adaptive experimental governance in a remote marine sanctuary with a wide range of stakeholder uses ranging from biodiversity conservation and endangered species protection to nearby oil and gas extraction. During interviews, participants were presented with scenario-based prompts derived from climate and ecological models (Appendix Figs. 3–4), which were used to guide discussion of potential responses and perceived constraints under projected environmental change. Our case contributes to the growing literature on remote marine protected areas. For example, Brooks et al. (2019) studied the Subantarctic Heard and McDonald Islands describing similar logistical, monitoring and governance challenges, and emphasizing the importance of experimental governance and adaptive management. Barragán Paladines & Chuenpagdee (2015) found that insufficient stakeholder inclusion may create management challenges that cannot be overcome by laws alone in the Galapagos Marine Reserve. Our study examines the unique context of a remote marine protected area where its nearest communities are among America’s largest cities (Houston and Galveston), alongside seaside small towns. Importantly, this combination of offshore remoteness and proximity to highly industrialized and urbanized coastal regions creates distinct challenges for fostering stakeholder inclusion and dialogue. Unlike more accessible marine protected areas, engagement at Flower Garden Banks must be actively cultivated across physical and perceptual distance, reinforcing the central motivation of this study. The diversity in coastal development of adjacent communities presents a challenge for building public awareness and engagement for the sanctuary due to the diversity of economic interests, cultural identities, and connections to the ocean that shape how people perceive and value the sanctuary. Much of what we know about coral reefs next to major cities comes from studies of urban reefs in East and Southeast Asia (Heery et al., 2018) and from research on resilient “urban corals” in the Port of Miami (Oceanographic & Meteorological Laboratory, 2025), yet Flower Garden Banks is unique in that all experiences with its reefs take place far offshore, with public awareness and engagement mediated through distant coastal communities rather than direct, everyday contact.

An important contribution of the research design was the integration of model-based climate scenarios into stakeholder interviews as a structured elicitation approach. Rather than relying solely on abstract discussion of climate change, respondents reacted to a common set of plausible ecological futures involving warming, bleaching, and fish biomass changes. This helped move interviews beyond general perceptions of environmental change toward discussion of governance capacities, barriers, and possible responses under specific projected conditions. The value added by this design lies not in testing stakeholder responses to multiple competing scenarios, which was beyond the scope of this study, but in using modeled futures to create a shared reference point for dialogue. In this sense, the modeling did not merely precede the interviews; it structured the inquiry itself. By applying our framework, collaborative adaptive experimental governance, specifically the concepts of dialogue and inclusion and barriers to responses to environmental change, we show that Flower Garden Banks National Marine Sanctuary has the core elements of collaborative adaptive experimental governance, while also facing challenges. Its foundation rests on federal laws such as the National Marine Sanctuaries Act and the Endangered Species Act, which shape the sanctuary’s mission and require management plans to undergo public review every five years. This process embeds opportunities for stakeholder participation, learning, and regulatory change. Prior studies note that legislation alone does not guarantee success; effective governance depends on coordination across scales and agencies (Ayers and Kittinger, 2014; Dunning, 2018; Kittinger et al., 2014). Other studies have shown that the marine sanctuary is not a successful governance arrangement solely because of these laws, but rather because the multiple scales of government and agencies that share responsibilities for management coordinate their jurisdictions (Kittinger et al., 2014). Research on the Galápagos Marine Reserve similarly shows that gaps between legal frameworks and social realities can hinder management (Barragán Paladines & Chuenpagdee, 2015), both of which are also relevant for Flower Garden Banks. Stakeholder inclusion is key to bridging these gaps. Our findings show how coordination among NOAA sanctuary managers, the Gulf Council, and the Bureau of Ocean Energy Management exemplifies coordination across jurisdictions needed to manage ecosystems effectively. For example, the sanctuary’s boundary expansion process involved coordination among NOAA, the Gulf Council, and BOEM to align conservation goals with existing oil and gas regulations, enabling expansion without significant stakeholder conflict. Likewise, stakeholder inclusion in Flower Garden enables stakeholders to step into important roles in education and public engagement that increase ownership of the sanctuary while building capacity for implementing its formal responsibilities. This is reflected in SAC-led initiatives such as Seaside Chats, aquarium partnerships, and stakeholder-led outreach efforts that extend the sanctuary’s engagement capacity.

The heart of collaborative adaptive experimental governance in this and others in the marine sanctuary system is the SAC. Importantly, the SAC operates within a broader federal advisory tradition under FACA, which provides procedural structure for openness, representation, and legitimacy. In this sense, the SAC is not unique simply because it is advisory, but because of how those advisory functions are used in practice to facilitate sustained dialogue among stakeholders with divergent interests. Our findings can also be interpreted through collaborative governance theory. For example, long-standing relationships among stakeholders reflect favorable starting conditions; the Sanctuary Advisory Council reflects institutional design; and repeated dialogue, working groups, and joint fact-finding reflect core collaborative processes as described by Ansell and Gash (2008). Viewed this way, our findings largely support established collaborative governance theory, while suggesting that climate scenario elicitation and prospective experimentation may extend those models in social-ecological systems facing environmental change. This formalizes stakeholder inclusion through the institution, granting seats to the entire range of sanctuary users, including oil and gas, anglers, recreational divers, local community groups, and conservation groups. Deliberative processes, where conversations occur over possible decisions, inform decision-making from NOAA sanctuary managers. The recommendations of the SAC carry weight with managers, even though their role is advisory. Other studies have identified the SAC as the heartbeat of public engagement, science, and capacity-building efforts in other sanctuary systems such as the Florida Keys (Morris et al., 2024). Research shows that advisory institutions can help trust in marine governance when these institutions are seen as legitimate and genuinely responsive to the needs of participants (Armitage et al., 2011; Turnhout et al., 2020). This is all the more important when stakeholders have such divergent interests as heavy industry and ecotourism. In U.S. public land management, agencies like the U.S. Forest Service have long managed National Forests for multiple uses, a system that balances recreation, conservation, and industry (Clarke and McCool, 1996). Flower Garden Banks demonstrates how similar coexistence is possible in the marine realm, where oil and gas operations, fishing, and biodiversity protections overlap and are negotiated by stakeholder inclusion. Comparative research across terrestrial and marine multiple-use contexts could yield valuable insights for governance.

There’s no better example of collaborative process in the sanctuary than the SAC’s Boundary Expansion Working Group, which held over 20 meetings to ensure expansion matched the interests of oil and gas, commercial fisheries, and recreational users. Stakeholders co-creating these regulations, changing regulations over time, in a setting where all stakeholders are included, embodies collaborative adaptive experimental governance, and is noteworthy for the legitimacy of the rules they create and the processes altogether. Other studies from other marine protected areas for case sites such as Indonesian marine protected areas, Hawaii’s Papahānaumokuākea, and Australia’s Great Barrier Reef have shown that stakeholder inclusion has a strong relationship to a protected area’s legitimacy (Day, 2008; Dunning, 2021b; Vaughan and Ardoin, 2014). One explanation of the high levels of legitimacy perceived by stakeholders for the sanctuary is the importance of relationships, with most respondents mentioning handshakes, face-to-face agreements, and similar interactions with sanctuary managers and stakeholders. An important implication of our findings is that stakeholder collaboration in Flower Garden Banks appears to rest not only on formal participatory structures, but also on long-standing relational and institutional foundations. In both oil and gas and fisheries, collaboration was embedded within pre-existing legal authorities, repeated interactions among actors, and informal norms of trust and reciprocity. This suggests that the effectiveness of multi-stakeholder governance may depend not simply on creating participatory forums, but on the historical relationships and institutional pathways upon which those forums are built. It’s important to note that while this study does not evaluate implemented ecosystem experiments directly, it examines an earlier and necessary stage of experimental governance: the use of modeled futures and stakeholder dialogue to identify institutional conditions, barriers, and capacities that shape whether experimentation is possible. In this sense, the contribution of this study lies less in evaluating experimentation outcomes than in examining governance conditions that may enable or constrain future experimentation.

Our study identified key blockages for sanctuaries responding to environmental change, including funding shortfalls, logistical barriers because of sanctuary remoteness, a lack of public knowledge about the sanctuary, low levels of trust in government, and possible bureaucratic slowdowns. One key challenge at the top of the list of priorities for stakeholders was that the distance to nearby communities, as well as the range of cities, small towns, and everything in between, made it difficult to raise public awareness that there is a sanctuary offshore. This brought about an out of sight out of mind mentality in nearby communities that could make responding to future environmental change difficult. Other studies have suggested that marine protected areas might become significant sources of local cultural pride and identity, such as the Phoenix Islands Protected Area and the people of Kiribati (Gruby et al., 2017). In our study, stakeholders are beginning to address this issue by partnering with aquariums and developing interactive exhibits at popular cruise terminals that attract thousands of visitors each month. These partner organizations, acting as mediators between the public, science, and policy, have been described elsewhere as boundary organizations and are known routes to increasing public awareness of conservation efforts (Cash et al., 2003).

From a scientific management perspective, one unifying issue across all five blockages includes the use of our climate scenario to elicit widespread stakeholder demand for funding for additional monitoring efforts and restoration science to inform management, such as genetic studies, mesophotic habitat research, and fish population dynamics. This situation, where there is political will to enact science-based management and restoration, but material limitations have been shown elsewhere in the literature (Ansell and Bartenberger, 2016). It could be possible that increasing public awareness of the sanctuary could lead to additional public will to increase sanctuary funding for such efforts. Like other studies of environmental governance, ours align with broader critiques across species and systems that show while collaborative processes have their benefits, bureaucracy, lack of awareness, long time periods to arrive at decisions, and inertia present challenges for these types of systems (Morrison et al., 2019).

## Conclusion

Remote marine sanctuaries with well-preserved yet sensitive biodiverse systems, exemplified by Flower Garden Banks National Marine Sanctuary, have unique challenges. Many may never visit these protected areas themselves, and the public may know little about them. Flower Garden Banks demonstrates how collaborative adaptive experimental governance balances stakeholder uses ranging from extractive industrial stakeholders in oil and gas and fisheries, to biodiversity conservation interests such as the diving industry and the aquarium, carrying on a long tradition of multiple-use management exemplified in American public land and waterway management agencies. The unique collaborative institutions like the SAC are essential to managing experimental decision-making processes as well as management endeavors like the sanctuary’s recent expansion. Environmental change, such as rising sea surface temperatures, changing movement ecology for fish, and coral bleaching, present increasing risks for stakeholders. Innovations for awareness building through citizen science, community-facing exhibits, and partnerships alongside continued use of collaborative adaptive experimental governance will ensure these unique reefs endure.

More specifically, our findings suggest several sector-specific recommendations. For NOAA and sanctuary managers, priorities include expanding monitoring capacity, investing in restoration readiness, and strengthening boundary-spanning outreach with coastal communities. For offshore energy actors and the Bureau of Ocean Energy Management, maintaining collaborative working groups and building on existing coordination mechanisms, such as No Activity Zones, may help sustain coexistence under future climate pressures. For fisheries actors and the Gulf of Mexico Fishery Management Council, continued use of collaborative processes to address emerging ecological change and regulatory overlap will remain important. For coastal communities and public-facing partners such as aquariums and schools, expanding education, citizen science, and place-based awareness efforts may strengthen long-term public support for sanctuary stewardship.

## Data Availability

The data supporting this study consist of confidential human-subject interview materials and cannot be publicly archived due to Institutional Review Board restrictions.

## Appendix

Appendix 1. Interview Questions

1. What are the primary ways you interact with the reefs (e.g. manager, user, business, etc.)?
2. What type of management do you do on the reefs of the FGBMS?
3. Can you describe opportunities for talking to others who use and manage the reef?
4. Can you describe how stakeholders are or are not included in decision-making in the management of the FGBMS?
5. Can you describe the way that your organization/user group is invited to play a role in management?
6. Scenario(s): Flower Garden Banks in the present day.
7. What are the blockages/challenges to respond to climate impacts?
8. What are the partnerships to respond to climate impacts?
9. Likert scale 1-5: In the management of the reefs of the FGBNMS, experimentation of new management options is possible due to climate change? A. Strongly agree, B. Agree, C. Neutral, D. Disagree, E. Strongly disagree
10. Can you explain how experimentation is possible due to climate change?
